# Crosstalk between Medulloblastoma Cells and Endothelium Triggers a Strong Chemotactic Signal Recruiting T Lymphocytes to the Tumor Microenvironment

**DOI:** 10.1371/journal.pone.0020267

**Published:** 2011-05-27

**Authors:** Vita S. Salsman, Kevin K. H. Chow, Donald R. Shaffer, Huseyin Kadikoy, Xiao-Nan Li, Claudia Gerken, Laszlo Perlaky, Leonid S. Metelitsa, Xiuhua Gao, Meena Bhattacharjee, Karen Hirschi, Helen E. Heslop, Stephen Gottschalk, Nabil Ahmed

**Affiliations:** 1 Center for Cell and Gene Therapy, Baylor College of Medicine, Houston, Texas, United States of America; 2 Texas Children's Cancer Center, Baylor College of Medicine, Houston, Texas, United States of America; 3 Department of Pediatrics, Baylor College of Medicine, Houston, Texas, United States of America; 4 Department of Pathology and Immunology, Baylor College of Medicine, Houston, Texas, United States of America; 5 Department of Medicine, Baylor College of Medicine, Houston, Texas, United States of America; University of Pennsylvania, United States of America

## Abstract

Cancer cells can live and grow if they succeed in creating a favorable niche that often includes elements from the immune system. While T lymphocytes play an important role in the host response to tumor growth, the mechanism of their trafficking to the tumor remains poorly understood. We show here that T lymphocytes consistently infiltrate the primary brain cancer, medulloblastoma. We demonstrate, both *in vitro* and *in vivo*, that these T lymphocytes are attracted to tumor deposits only after the tumor cells have interacted with tumor vascular endothelium. Macrophage Migration Inhibitory Factor (MIF)” is the key chemokine molecule secreted by tumor cells which induces the tumor vascular endothelial cells to secrete the potent T lymphocyte attractant “Regulated upon Activation, Normal T-cell Expressed, and Secreted (RANTES).” This in turn creates a chemotactic gradient for RANTES-receptor bearing T lymphocytes. Manipulation of this pathway could have important therapeutic implications.

## Introduction

Growing evidence indicates that cancer cells can only live and grow if they succeed in creating a niche that favors their survival and progression. [1] This microenvironment often includes vascular endothelial cells, supporting stromal cells as well as elements from the immune system. [2]–[5] T lymphocytes constitute one of the most important effector mechanisms of antitumor immunity and often contribute to the tumor microenvironment and their role remains controversial. [6] T lymphocytic infiltrates have been correlated with antitumor immunity and favorable outcomes in a number of tumors. [6]–[10] Other reports demonstrated the facilitative role of infiltrating T lymphocytes to the survival and progression of tumor cells. [11]–[13] These observations point out to the potential duality of the role of T lymphocytes in the tumor microenvironment: possibly attempting to cordon the tumor progression but also sustaining the tumor niche.

While there is substantial knowledge about the sequence of events creating the signals that drive leukocyte infiltration in models of inflammation and autoimmune disease, [14]–[24] little is known about the mechanistic events that attract T lymphocytes to the tumor microenvironment. Decoding such events would increase our understanding of the interaction between the tumor cells and tumor infiltrating T lymphocytes and help develop strategies to enhance or block this crosstalk. Growing evidence supports the essential and complex role of the vascular endothelium in tumor biology and cancer progression. [25]–[27] It is now evident that the interactions between vascular endothelial cells and the tumor microenvironment may regulate tumor progression in a tumor-type-specific manner. [25]

We show here, using medulloblastoma as a model, that T lymphocytes may only be attracted to tumor deposits if the tumor cells have interacted with the tumor vascular endothelium. *M*acrophage Migration *I*nhibitory *F*actor (MIF)” is the key primary chemokine molecule secreted by tumor cells which induces the potent T lymphocyte attractant “*R*egulated upon *A*ctivation, *N*ormal *T*-cell *E*xpressed, and *S*ecreted (RANTES)” from endothelial cells, which in turn creates a chemotactic gradient for RANTES-receptor bearing T lymphocytes. Manipulation of this pathway could have important therapeutic implications.

## Results

### T lymphocytes consistently infiltrate primary medulloblastoma

**Figure 1:**
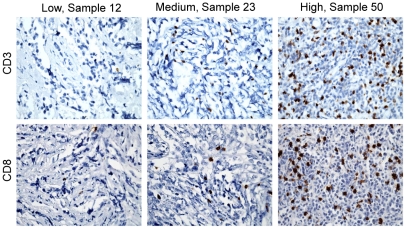
T lymphocytes consistently infiltrate primary medulloblastoma. Representative photomicrographs of CD3 IHC of human primary medulloblastoma demonstrating variable degrees of infiltration: modest perivascular pattern (≤1%; 15 of 20 tumors), moderate perivascular infiltration with varying degrees of parenchymal spill (>1–10%; 4 of 20 tumors) and uniform heavy perivascular and parenchymal infiltration (>10%; 1 of 20 tumors). Similarly, tumor sections from the same subjects were stained for CD8 using IHC and showed similar degrees and patterns of infiltration. Magnification x100.

T lymphocytes constitute one of the most important effector mechanisms of anti-tumor-immunity and often contribute to the tumor microenvironment. It is well established that T lymphocytic infiltrates are present in tumors outside the neuraxis, where they can have tumor inhibitory or promoting effects. [6]–[13] In contrast, less is known about these infiltrates in the context brain tumors. We therefore opted to study this in medulloblastoma, the most common pediatric brain tumor. We examined a series of primary medulloblastoma and found a T lymphocyte infiltration in 20 of 20 tumors examined using immunohistochemistry (IHC) for CD3. The degree of the T lymphocyte infiltrate varied between individual tumors and ranged between a modest perivascular pattern (≤1%; 15 of 20 tumors); moderate perivascular infiltration with varying degrees of parenchymal spill (>1–10%; 4 of 20 tumors) to a uniform heavy perivascular and parenchymal infiltration (>10%; 1 of 20 tumors). Similarly, tumor sections from the same subjects were stained for CD8 using IHC and showed similar degrees and patterns of infiltration (Figure 1).

### T lymphocytes migrate and localize to distant tumor deposits in the brain

**Figure 2:**
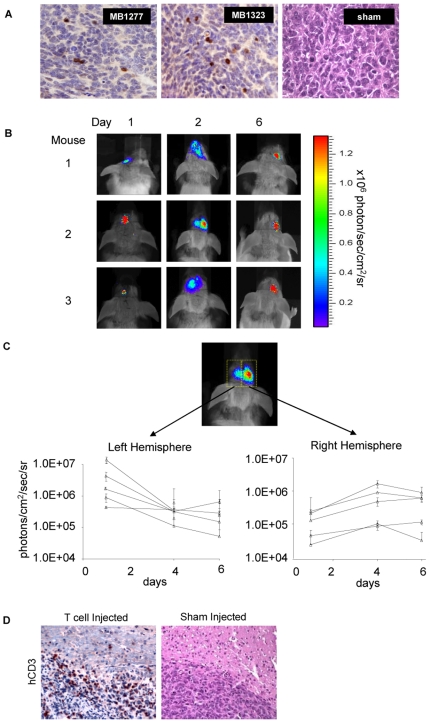
T lymphocytes migrate and localize to distant tumor deposits in the brain. (A) IHC for human CD3 in explants of tumors, propagated in the right cerebellum from subjects MB1277 and MB1323, 6 days after human T lymphocytes were injected into the ipsilateral caudate nucleus (x200). (B) Experimental tumors were established from the medulloblastoma cell line, Daoy, in the right caudate nucleus of SCID mice. Ten days later firefly luciferase-expressing T lymphocytes were stereotactically injected into the contralateral (left) caudate. T lymphocytes migrated within the brain to contralateral tumors in all experimental animals (n = 5) as judged by bioluminescence imaging. (C) The migration pattern of T lymphocytes was monitored over predefined gates placed over the left and right frontal lobes. Quantitative bioluminescence imaging indicated T lymphocyte efflux into the contralateral tumor gate in all experimental animals. (D) Immunohistochemistry for human CD3 performed on a subset of animals confirmed clustering of T lymphocytes at the tumor site.

We hypothesized that these infiltrates result from an innate avidity of T cells to the tumor cells and or the tumor complex. To test this hypothesis, we used a murine model in which human primary medulloblastoma (from subjects MB1277 and MB 1323) were orthotopically propagated in the right cerebellar cortex. Ten days after tumor establishment, mice were injected with T lymphocytes or sham injected into the right (ipsilateral) caudate nucleus. The injected T lymphocytes had a predominantly CD8-positive population. Six days later, the tumors were removed and examined by IHC for human CD3. Figure 2A shows that human CD3-positive cells could be detected only in animals receiving human T lymphocytes but not sham injected animals, suggesting that primary medulloblastoma can trigger T lymphocyte migration from distant sites in the brain. To confirm T cell migration in the Daoy human medulloblastoma model by bioluminescence imaging, we injected luciferase-expressing human T cells into the left caudate nucleus of mice with established, 10 day old Daoy tumors in their contralateral caudate nucleus. T lymphocytes migrated within the brain to contralateral tumors in all experimental animals as shown in Figures 2B and 2C. Immunohistochemistry using human CD3-specific antibodies demonstrated columns of T lymphocytes migrating along the corpus callosum, the only solid connection between both hemispheres, and their clustering at the tumor periphery as well as within the tumor itself (Figure 2D).

### T lymphocytes that migrated to tumors *in vivo* fail to migrate to tumor cells *in vitro*

**Figure 3:**
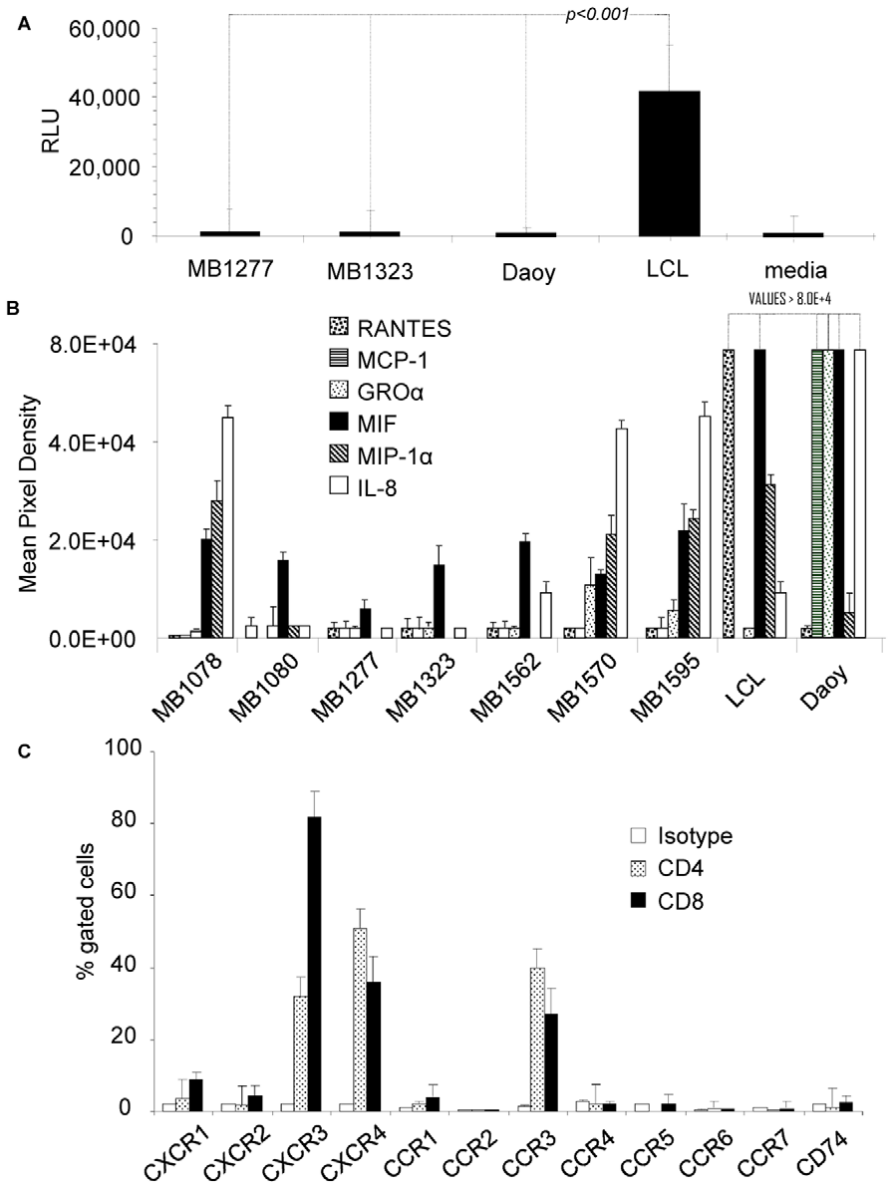
T lymphocytes that migrated to tumors *in vivo* fail to migrate to tumor cells *in vitro*. (A) T lymphocytes were placed in the upper wells of 4 µm transwell systems. Supernatants from primary medulloblastoma cells (MB1277 and MB1323), Daoy medulloblastoma cells, LCLs and unconditioned media were placed in the lower well. While LCL supernatants induced brisk T lymphocyte migration (p<0.001), their migration towards Daoy supernatants was comparable to that of media only. (B) Chemokine signatures of Daoy and 7 primary medulloblastoma culture supernatants. (C) Flowcytometry of CD4 and CD8 subsets to study the chemokine receptor expression pattern on T lymphocytes. No chemokine receptors corresponding to tumor-derived chemokines were detectable on the cell surface. This explained the failure of supernatants from tumor cells to attract T lymphocytes ex vivo.

T lymphocyte migration has generally been attributed to a response to a chemokine gradient generated by chemokine-secreting tumors. [28]–[32]. To examine the mechanism whereby tumor cells are attracting T lymphocytes, we performed a series of *ex vivo* transwell studies. Surprisingly, the same T lymphocytes that avidly migrated *in vivo* to tumor deposits failed to migrate *in vitro* when exposed to supernatants from cultures of the primary medulloblastoma samples in short-term culture or to the medulloblastoma cell line, Daoy (Figure 3A). Failure to migrate could not be explained by adverse *in vitro* cell culture conditions, since positive control B-lymphoblastoid cell line (LCL) supernatants induced brisk migration of T lymphocytes *in vitro*. As anticipated, no migration was seen in response to media alone.

To determine the mechanism responsible for failure of T lymphocyte migration to tumor cells *in vitro*, we characterized the chemokine profile of the tumor cells using a semiquantitative chemokine profiling system. We then used flow-cytometric analysis of T lymphocytes to determine their expression of the chemokine counter-receptors. Daoy medulloblastoma cells had a distinct chemokine signature, producing MCP-1; Gro-α; MIF; IL-8; Serpin E1; and IL-6 (Figure 3B and Figure S1). All 7 primary tumors secreted MIF, 4 tumors secreted IL-8, 3 tumors secreted MIP-1α and only one tumor secreted IL-13 and IL12. MB1277 and MB1323 secreted only MIF, the only molecule common to all samples tested. T lymphocytes, however, only expressed substantial levels of CXCR3, CXCR4 and CCR3 on their surface; CXCR1, CXCR2 and CCR1 were expressed by less than 10% of cells (Figure 3C). Hence T lymphocytes lacked measurable chemokine receptors that are cognate for the chemokines the tumor cells produce, explaining the observed lack of *in vitro* migration.

### Tumor cells trigger the secretion of the potent chemo-attractant: *R*egulated upon *A*ctivation, *N*ormal *T*-cell *E*xpressed, and *S*ecreted (RANTES) from the endothelium and induce avid T-cell migration *in vitro*

**Figure 4:**
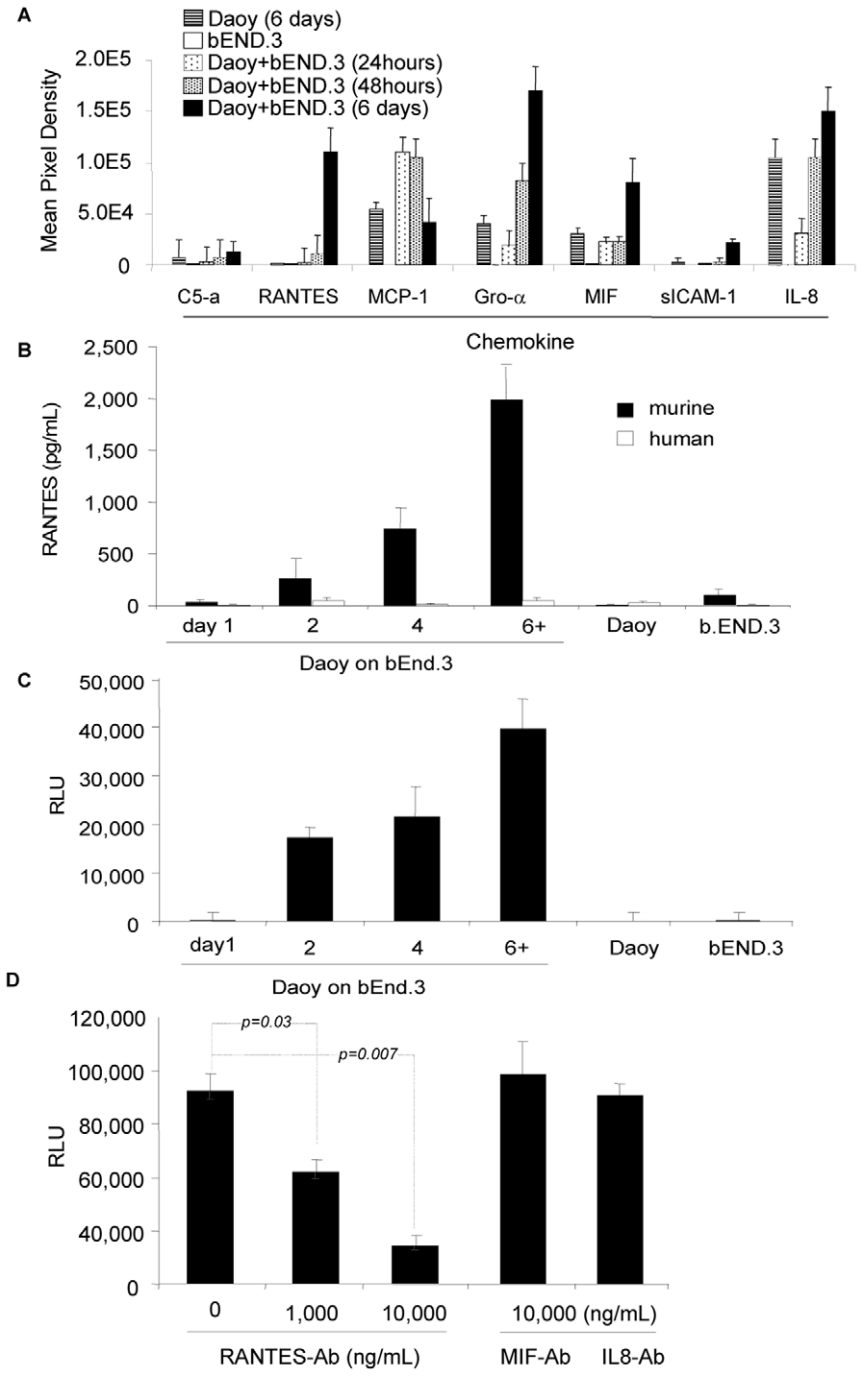
Supernatant from medulloblastoma and brain endothelial cell coculture induces avid T lymphocyte migration *in vitro* through the induction of RANTES. (A) Semiquantitative measurement of chemokines using protein profiler membranes showed that while RANTES was near undetectable in supernatant from bEND.3 or in supernatant from Daoy grown for 6 days, its secretion substantially increased as early as 48 hours of Daoy and bEND.3 coculture and rapidly increased over time to plateau after 6 days. (B) ELISA for murine RANTES quantitatively confirmed these results and determined that RANTES is endothelium- rather than tumor-derived. (C) Supernatants from Daoy and b.END.3 coculture system were used to test T lymphocyte migration in transwell systems. In contrast to supernatants from tumor or that from brain endothelium cells, supernatants of tumor and endothelium cocultures induced avid migration of T lymphocytes. (D) Using 1,000 ng/mL and 10,000 ng/mL of RANTES blocking Ab decreased T lymphocyte migration by 30 and 60 %, respectively (*p = 0.03* and *0.007*, respectively) confirming the dominant role of RANTES in triggering T lymphocyte migration. Blocking Ab to IL8 and MIF up to 10,000 ng/mL failed to counteract the migration to Daoy and bEND.3 coculture supernatants.

The discrepancy between the *in vivo* and *in vitro* migration of T lymphocytes, led us to hypothesize that successful migration occurred *in vivo* because primary tumor cells triggered a secondary, tumor-stroma derived, chemokine signal that was successfully recognized by T lymphocytes. Growing evidence supports an essential and complex role for the vascular endothelium in cancer biology and progression. [25] In particular, crosstalk between endothelial and tumor cells was shown to regulate tumor progression and resistance to anti-cancer therapies in various manners depending on the tumor type. [25]–[27] We therefore cocultured tumor cells with murine brain tumor endothelial cells (bEND.3) to more closely mimic our *in vivo* system. We characterized changes in the chemokine profile during the period of coculture. Supernatants from Daoy and bEND.3 cocultures contained a chemokine pattern that was different from those observed in either cell population alone. Distinctly, the coculture contained large amounts of RANTES, which were detectable after 48 hours and reached a plateau after 6 days (Figure 4A and Figure S2). This effect was not contact-dependent since it was preserved when tumor and endothelial cells were separated by a 0.2 µm pore membrane. Other chemokines/chemo-attractant molecules that were present in low concentrations but progressively increased in coculture included C5-a, MCP-1, Gro-α, sICAM-1, MIF and IL-8. These molecules, unlike RANTES, were also present at low concentrations in Daoy only supernatants and increased to higher levels in proportion to the degree of confluency of the Daoy cells.

Next, we determined the source of RANTES in the tumor/endothelium coculture. At the protein level, murine RANTES shows 88% homology with human RANTES and exhibits species cross reactivity on human and mouse cells. [33], [34] The RANTES probe in our chemokine protein multiplex assay cross reacted with both human and murine RANTES. Therefore we determined whether the RANTES we detected was human- (tumor cell) or murine- (endothelial cell) derived by re-measuring RANTES levels using a murine-specific ELISA. Our species-specific assay showed high activity of murine endothelium-derived protein but not of its human counterpart (Figure 4B).

To test if the signal derived from the tumor–endothelial coculture system attracts T lymphocytes: we collected these supernatants daily and used them to test T lymphocyte migration in a transwell system. Culture supernatants from tumor or endothelial cells alone grown for six days induced minimal migration of T lymphocytes. By contrast, we observed avid migration of T lymphocytes when we used supernatants from the combination of tumor and endothelial cells. (Figure 4C) The degree of migration paralleled the concentration of RANTES protein in the supernatants, becoming detectable after 48 hours of coculture, and progressively increasing to plateau from day 6. To determine the role of RANTES and other chemokines in inducing T lymphocyte migration, we incubated 6 day Daoy and bEND.3 coculture supernatants with RANTES-blocking antibody (Ab) or IL8- or MIF-blocking Ab, as controls. RANTES Ab blocked the migration to the coculture supernatant by up to 60% while control IL8- and MIF-Ab failed to do so (Figure 4D).

### Tumor-derived “*M*acrophage Migration *I*nhibitory *F*actor (MIF)” is the primary tumor chemokine signal triggering RANTES release from the endothelium

**Figure 5:**
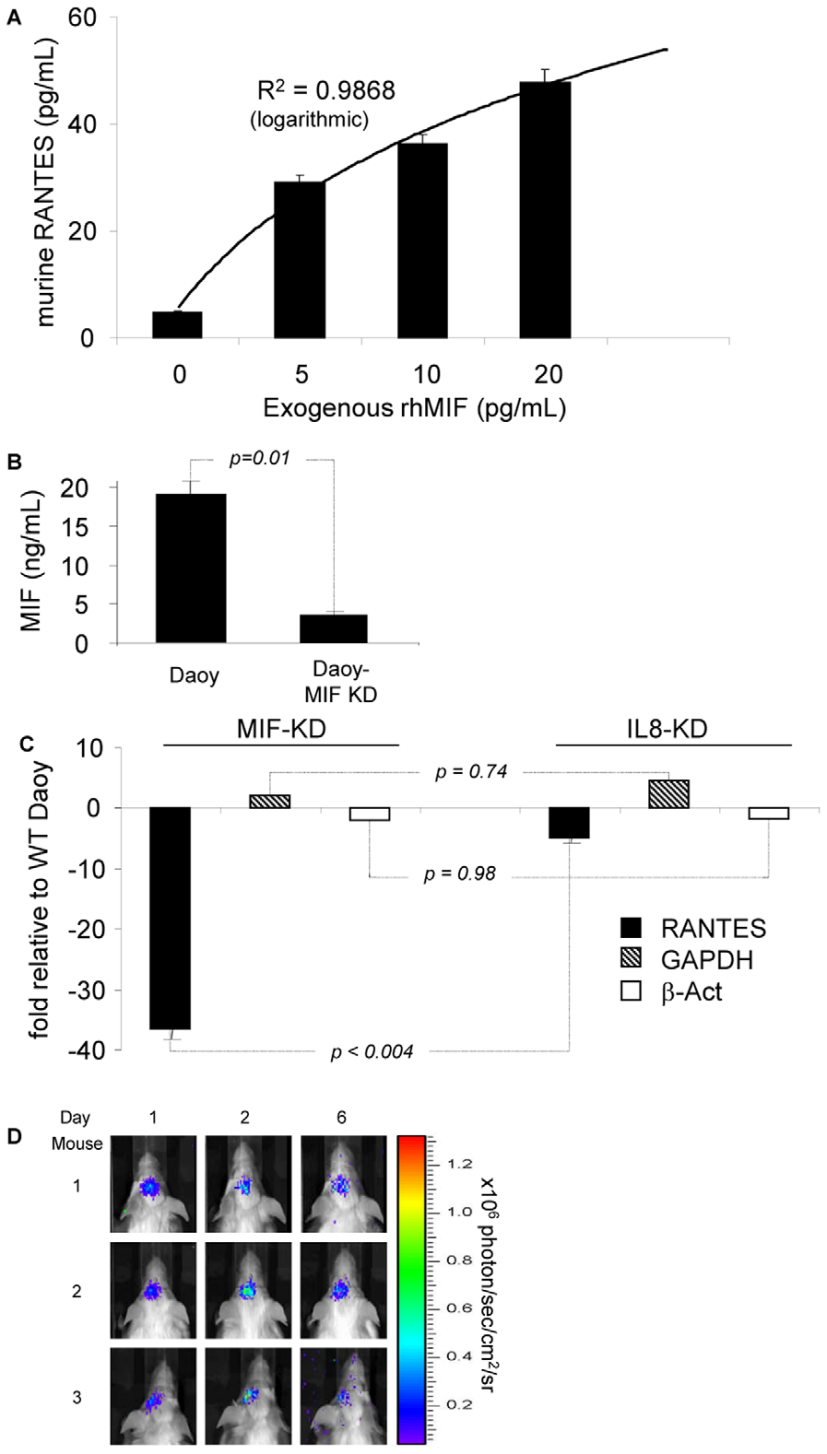
The primary medulloblastoma chemokine signal triggering the release of RANTES is mediated by MIF. (A) Increasing concentrations of exogenous recombinant human (rh)MIF added to 72 hour cultures of bEND.3 cells induced the release of murine RANTES in a rhMIF-dose-dependent manner. (B) Supernatants from cell cultures analyzed for the production of MIF showing that Daoy cells transduced with a MIF-specific shRNA encoding construct (MIF-KD Daoy) secreted substantially less MIF (<20%) compared to the parental Daoy cell line. (C) RNA was extracted from tumors established from Daoy, MIF-KD Daoy and IL-8-KD Daoy and analyzed for the presence of RANTES as well as GAPDH and β-Actin (controls). While IL-8-KD Daoy tumors showed minimal inhibition of RANTES RNA (4.8 fold decrease), MIF-KD Daoy tumor RNA showed a 38 fold decrease in expression compared to the parental cell line Daoy. Both house-keeping genes were minimally altered. (D) MIF-KD Daoy cells induced similar size tumors in comparison to parental Daoy cells but failed to induce T lymphocyte migration *in vivo*.

Our chemokine array analysis had revealed that all 7 primary medulloblastomas and Daoy secreted MIF, and that neither secreted RANTES (Figure 3B and Figure S1 and S2). To investigate if MIF acts directly on endothelial cells to release RANTES, we incubated bEND.3 cells with increasing concentrations of recombinant human MIF (rhMIF), a molecule that is 90–98% homologous to murine MIF and biologically active on both human and murine cells. [14], [35] Using a murine ELISA, we observed that supernatants from such cultures contained RANTES in a MIF-dose-dependent concentration (Figure 5A). We thus concluded that, *in vitro*, MIF can directly trigger the release of RANTES from endothelial cells.

To further confirm *in vivo* that MIF was necessary for triggering RANTES production from endothelial cells, we generated MIF-knock down (KD) Daoy medulloblastoma cells using a retroviral vector encoding a MIF-specific shRNA. The MIF-specific shRNA reduced MIF production by 80% in comparison to the parental cell line (Figure 5B). To corroborate the link between expression of MIF and RANTES *in vivo*, we established tumors from the parental cell line Daoy, from MIF-KD Daoy cells, and from IL-8-KD Daoy cells (control). RNA was extracted from the tumor and the adjacent brain matter analyzed by qPCR for RANTES and two housekeeping genes (GAPDH and β-actin). While IL-8-KD Daoy tumors induced murine RANTES RNA levels similar to those of un-manipulated Daoy, MIF-KD Daoy tumor RNA showed a 38-fold decrease in RANTES expression compared to parental Daoy tumors. Control gene expression was similar among Daoy, MIF-KD and IL8-KD Daoy (Figure 5C). Hence, MIF-KD tumor cells failed to induce murine RANTES *in vivo*; confirming that MIF released by tumor cells is the primary chemokine that triggers the release of RANTES from endothelial cells.

Finally, we studied, *in vivo*, the requirement of the MIF-RANTES axis for T lymphocyte migration to the tumor site. Ten days after establishing tumors derived from MIF-KD Daoy in the right caudate nucleus of mice, we stereotactically injected firefly luciferase-expressing T lymphocytes into the left caudate nucleus. Histological examination of a subset of animals showed that Daoy and MIF-KD Daoy xenografts had similar volumes (not shown). Firefly luciferase-expressing T lymphocytes that migrated briskly to established Daoy tumors failed to migrate to the contra-lateral MIF-KD Daoy tumors (Figure 5D). IHC of tumors explanted from the right hemispheres confirmed the absence of T lymphocytes.

## Discussion

We have shown that the interaction between medulloblastoma cells and their associated endothelium triggers T lymphocyte migration into the tumor microenvironment. Medulloblastoma cells constitutively release the chemokine MIF which itself cannot recruit T lymphocytes, but triggers the release of a second chemokine, RANTES, from endothelial cells in the tumor stroma. This in turn attracts T lymphocytes since they express RANTES-cognate chemokine receptors. [36] We thus demonstrate that ‘cross-talk’ between the tumor cells and endothelium creates a chemotactic gradient triggering T lymphocyte migration to the tumor microenvironment.

Leukocyte migration to disease sites has been extensively studied in research models of inflammation and autoimmune diseases. [14]–[24] These studies have shown that in the presence of chemokines induced by the pathological process, leukocytes are activated and directed out of the vasculature into the sites of tissue injury. The role of cells in the tumor microenvironment, including endothelial cells, in creating a gradient for leukocyte migration is well characterized in these models. [14]–[24] In contrast, little is known about their role in the process of T lymphocyte migration in the context of malignancies. Decoding such mechanistic events would increase our understanding of T lymphocyte migration to the tumor microenvironment.

Our work describes a previously uncharacterized dimension to the phenomenon of intra-parenchymal T lymphocyte migration that involves a third party, the tumor endothelium. Coculture of medulloblastoma tumor cells and endothelial cells resulted in a new chemokine signature dominated by the progressive secretion of endothelium- rather than tumor-derived RANTES, a strong T lymphocyte chemo-attractant that is not produced by either cell type culture alone. Since CCR3 is strongly expressed on migrating T lymphocytes, we suspected that RANTES was the major chemoattractant of T lymphocytes to the tumor microenvironment. This was confirmed by the observed inhibition of T lymphocyte migration after RANTES neutralization.

While both MIF and IL-8 were secreted by the majority of primary tumors as well as by Daoy cell line, T lymphocytes migrated to two different experimental tumors derived from primary cells that secreted only MIF. MIF, a chemokine-like inflammatory mediator, triggers leukocyte recruitment by binding to CXCR2 and CXCR4 [37], [38]. While CXCR4 was expressed on T lymphocytes tested in this study, its chemotactic effect is contingent on forming heterodimers with cell surface-expressed CD74. [39] The latter was undetectable on the T lymphocytes, explaining their failure to migrate to MIF in transwell systems. We further demonstrated that knocking down MIF but not IL-8 in the tumor cell line results in failure of T lymphocytes to migrate to contralateral tumor deposits. Furthermore, RNA extracted from established tumors showed substantial inhibition of RANTES in MIF-KD explants. This indicates that MIF, derived from tumor cells, is responsible for RANTES production by endothelial cells and subsequently, T lymphocyte migration.

**Figure 6:**
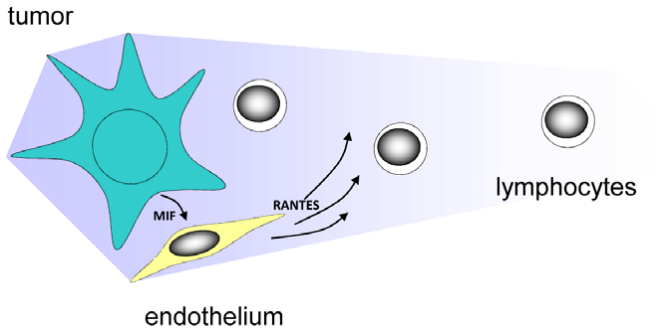
Proposed Model of T Lymphocyte Migration to Medulloblastoma Cells. MIF secreted by medulloblastoma cells induce endothelial cells to produce RANTES, which triggers the migration of T lymphocytes to the tumor microenvironment.

The vast majority of human chemokine molecules have a high degree of amino acid sequence homology to their murine counterpart. [14], [34], [35], [40]–[44] These molecules exhibit complete cross-species activity on human and mouse cells but, depending on the antibody used for testing, could be immunologically distinct. [14], [35], [40], [41] We thus took advantage of the xenogenic setup to determine the source of RANTES using both human and murine specific ELISA that employ antibodies specific to a non-conserved epitope on the molecule. To exclude the possibility that the observed induction of RANTES was due to a xenogeneic effect, we repeated the same experiment using co-culture of Daoy cells with primary Human Brain Microvascular Endothelial cells (HBMEC) and obtained similar results to those obtained with murine endothelial cells. (Figure S3) We thus propose a model of T lymphocyte migration as a result of the interaction between the tumor and endothelium as outlined in Figure 6.

Rubin et al. has shown previously that endothelial cells attract medulloblastoma cells through the CXCL12/CXCR4 axis and that this interaction is critical for tumor establishment. [45] Our results now indicate that the tumor cell/endothelial cell interactions results in recruitment of T lymphocytes, which have the potential to further promote tumor growth. Indeed, on examining a number of primary medulloblastomas, we found an infiltrate of T lymphocytes that was typically perivascular with various degrees of parenchymal involvement. This finding has implication for how tumor niches are formed and suggests a ‘domino effect’ in which the recruitment of one cell type triggers the recruitment of another.

Thus, interventions that inhibit the effects of MIF (to counteract the primary tumor-derived signal) [38] and or RANTES (to counteract the endothelium derived signal), or block their cognate receptors could possibly offset tumor growth by altering the tumor-induced niche. Conversely, the innate avidity of T lymphocytes to the tumor microenvironment could be exploited therapeutically by redirecting their specificity towards tumor-restricted antigens. Studies to test these therapeutic interventions by our group are currently underway.

In summary, we have shown that the crosstalk between tumor and endothelial cells creates an effective chemotactic gradient triggering the *in vivo* migration of T lymphocyte to the tumor microenvironment. Manipulation or exploitation of these interactions may be of benefit to tumor control.

## Materials and Methods

### Blood donors, primary tumor cells and cell lines

Studies were performed on Baylor College of Medicine IRB-approved protocols and informed written consent was obtained from all donors. The medulloblastoma line Daoy was purchased from ATCC (Manassas, VA). All cell lines were grown in DMEM (Invitrogen, Carlsbad, CA) with 10% fetal calf serum (FCS; HyClone, Logan, UT), with 2 mM GlutaMAX-I, 1.5 g/L sodium bicarbonate 0.1 mmol/L and 1.0 mmol/L sodium pyruvate (Invitrogen). T lymphocytes derived from PBMCs were activated on CD3 antibody-coated plates and were expanded in IL-2 (100U/mL)-containing RPMI 1640 with 10% FCS and 2 mM GlutaMAX-I. Primary Human Brain Microvascular Endothelial Cells (HBMECs) were obtained from CellScience (Carlsbad, CA) and were cultured in Endothelial Cell Medium, obtained from the same vendor.

Tumor tissues were processed aseptically, and primary cell cultures were initiated using the supplemented DMEM. Cells were used within three days of plating.

### Retroviral construct, retrovirus production and transduction of T lymphocytes

We used an SFG retroviral vector containing an eGFP-firefly luciferase fusion gene in order to generate firefly luciferase expressing T lymphocytes for the *in vivo* study. [46], [47] To produce retroviral supernatant, 293T lymphoctyes were cotransfected with retroviral vector containing plasmid, pEQ.PAM-E plasmid encoding the sequence for MoMLV gag-pol, and plasmid pMEVSVg containing the sequence for VSV-G, using GeneJuice transfection reagent (EMD Biosciences, San Diego, CA) [40]. Supernatants containing the retrovirus were collected 48 and 72 hours later. VSV-G pseudotyped viral particles were used to transduce the FLYRD18 producer cell line for the production of RD114 pseudotyped viral particles [41].

T lymphocytes were transduced with retroviral vectors as described. [46], [47] Briefly, peripheral blood mononuclear cells (PBMC) were isolated by Lymphoprep gradient centrifugation. 5×10^5^ PBMC per well of a 24-well plate were activated with OKT3 (OrthoBiotech, Raritan, NJ) at a final concentration of 1 µg/mL. On day 2, recombinant human IL-2 (Chiron, Emmeryville, CA) was added at a final concentration of 50 units/mL, and on day 3 cells were harvested for retroviral transduction. For transduction, we pre-coated a non-tissue culture treated 24-well plate with a recombinant fibronectin fragment (FN CH-296; Retronectin; Takara Shuzo, Otsu, Japan). Wells were washed with phosphate-buffered saline (PBS; Sigma, St. Louis, MO) and incubated twice for 30 minutes with retrovirus. Subsequently, 3×10^5^ T lymphocytes per well were transduced with retrovirus in the presence of 50 units IL-2 per mL. After 48–72 hours, cells were removed and expanded in the presence of 50–100 units IL-2 per mL for 10–15 days prior to use.

### Orthotopic xenogenic SCID mouse model of medulloblastoma

All animal experiments were conducted on a protocol AN3949 approved by the Baylor College of Medicine Institutional Animal Care and Use Committee (IACUC). Recipient NOD-SCID mice were purchased from Taconic (C.B-*Igh-1^b^*/IcrTac-*Prkdc^scid^*; FOX CHASE CB-17 SCID™ ICR; Taconic, Hudson, NY). Male 9 week to 12 week-old mice were anesthetized with rapid sequence inhalation isofluorane (Abbot Laboratories, England) followed by an intraperitoneal injection of 225–240 mg/kg Avertin® solution and then maintained on isofluorane by inhalation throughout the procedure. The head was shaved, then the mice were immobilized in a Cunningham™ Mouse/Neonatal Rat Adaptor (Stoelting, Wood Dale, IL) stereotactic apparatus fitted into an E15600 Lab Standard Stereotactic Instrument (Stoelting), then scrubbed with 1% povidone-iodine. A 10 mm skin incision was made along the midline. The tip of a 31G ½ inch needle mounted on a Hamilton syringe (Hamilton, Reno, NV) served as the reference point. A 1 mm burr-hole was drilled into the skull, 1 mm anterior to and 2 mm to the right of the bregma. Daoy cells (2.5×10^5^ in 2.5 µL) were injected 3 mm deep to the bregma, corresponding to the center of the right caudate nucleus over 5 minutes. The needle was left in place for 3 minutes, to avoid tumor cell extrusion, and then withdrawn over 5 minutes. Ten days after tumor cell injection, animals received 2×10^6^ eGFP.Firefly-luciferase-expressing T lymphocytes in 2.5 µL at the same tumor coordinates in the contralateral caudate nucleus (Bregma +1 mm anterior; +2 mm to the left of the midline, +3 mm deep). The incision was closed with 2–3 interrupted 7.0 Ethicon® sutures (Ethicon, Inc. Somerville, NJ). A subcutaneous injection of 0.03–0.1 mg/kg buprenorphine (Buprenex® RBH, Hull, England) was given for pain control. The primary medulloblastoma xenograft model was described previously. [48]

### Bioluminescence imaging

Isofluorane anesthetized animals were imaged using the IVIS® system (IVIS, Xenogen Corp., Alameda, CA) 10 minutes after 150 mg/kg D-luciferin (Xenogen) was injected intraperitoneally. [49] The photons emitted from luciferase-expressing cells within the animal body and transmitted through the tissue were quantified using "Living Image", a software program provided by the same manufacturer. A pseudo-color image representing light intensity (blue least intense and red most intense) was generated and superimposed over the grayscale reference image. Animals were imaged after injections daily. They were regularly examined for any neurological deficits, weight loss or signs of stress and euthanized according to pre-set criteria, in accordance the Baylor College of Medicine's Center for Comparative Medicine guidelines.

### Immunohistochemistry (IHC)

Mice were euthanized by CO2 inhalation and fixed with intra-cardiac perfusion of 4% paraformaldehyde. The brain tissue was post-fixed overnight and embedded in paraffin, and histology was performed on 10-µm serial horizontal sections. Tissue sections were stained by a standard hematoxylin and eosin technique. Human CD3 expression in T lymphocytes was detected by human CD immunohistochemistry. The same methodology was used to stain primary medulloblastoma samples.

### Transwell migration assays

T lymphocyte migration towards tumor chemokines was measured *in vitro* through the use of a transwell migration assay. Firefly luciferase expressing T lymphocytes (1×10^5^ in 100 µL serum-free media) were placed in the top insert chamber of 4µm transwell migration plate (Corning Inc., Corning, NY). The lower chamber was filled with 600 µL of either control medium, supernatant from various cell lines, or medium conditioned with a specific chemokine. After a 3 hour incubation at 37°C, the medium from the lower chamber was collected and bioluminescence was measured for 30 seconds after 15 second incubation with 150 µg D-luciferin (3010 Luminometer, BD Biosciences, San jose, CA).

### Analysis of chemokine production

The detection and quantification of chemokine production from tumor cells and endothelial cells was done using a cytokine array kit (Human and Murine Proteome Profiler^TM^ array, R&D Systems, Minneapolis, MN). Supernatant from tumor cells alone, endothelial cells alone, and both cell lines in co-culture were collected at various time points and tested for 36 different chemokines per the manufacturer's instructions.

Supernatant from the co-culture of endothelial cell lines and tumor cell lines were collected at various time points and tested by cytokine-specific ELISA per the manufacturer's instructions (R&D Systems, Minneapolis, MN).

### Flowcytometry

For all flowcytometric analyses, a FACScalibur instrument (BD, Becton Dickinson, Mountain View, CA) and CellQuest software (BD) were used. Data analysis was done on >10,000 events; in all cases negative controls included isotype antibodies. Cells were washed once with PBS containing 2% FBS and 0.1% sodium azide (Sigma; FACS buffer) prior to addition of antibodies. After 15 to 30 minutes of incubation at 4oC in the dark the cells were washed once and fixed in 0.5% paraformaldehyde/FACS buffer prior to analysis. T lymphocytes were analyzed with anti-CD8 FITC, -CD4 PE, and -CD3 PerCP as well as the chemokine receptors, CXCR1, CXCR2, CXCR3, CXCR4, CCR1, CCR2, CCR3, CCR4, CCR5, CCR6, CCR7 and CD74. All monoclonal antibodies were obtained from BD Biosciences, Palo Alto, CA.

### Generation of knockdown medulloblastoma lines

MIF knockdown (KD) and IL-8-KD Daoy cells were generated by transducing Daoy cells with RD114 pseudotyped retroviral vectors encoding shRNAs for MIF or IL8 (pRS shRNA; 4 shRNAs per target gene; Origene; Rockville, MD). Transduced cells were selected with puromycin, and MIF or IL-8 production was measured by ELISA (R&D Systems, Minneapolis, MN). Daoy cell lines with the greatest decreased of MIF (81% of controls) or IL-8 (78% of controls) production were used in subsequent experiments.

### RNA extraction and analysis

RNA was isolated from explanted Daoy and KD Daoy xenografts. Total RNA concentrations were measured using the Nanodrop 1000 spectrophotometer (Nanodrop Scientific, Wilmington, DE). RNA integrity was checked in electrophoresis using 2% agarose gel with 6% formaldehyde. The human and murine chemokine and chemokine receptor arrays were performed by SABiosciences Inc (Frederick, MD) using PAHS-022 (human) and PAMM-022 (mouse) chemokine and receptor arrays.

### Statistical analysis

For the bioluminescence experiments, intensity signals were log-transformed and summarized using mean ± SD at baseline and multiple subsequent time points for each group of mice. Changes in intensity of signal from baseline at each time point were calculated and compared using paired t-tests or Wilcoxon signed-ranks test.

## Supporting Information

Figure S1 **A protein profiler system was used to characterize the chemokine pattern specific to Daoy medulloblastoma cells which had a distinct chemokine signature, producing MCP-1; Gro-α; MIF; IL-8; Serpin E1; and IL-6 [quantified in** **Figure 3B** **].**
(TIF)

Figure S2 **Chemokine protein profiler membranes showed that while RANTES was near undetectable in supernatant from bEND.3 or in supernatant from Daoy, its secretion substantially increased as early as 48 hours of Daoy and bEND.3 coculture and rapidly increased over time to plateau after 6 days.** Other chemokines/ chemo-attractant molecules that were present in a low concentration but progressively increased in coculture were also present at low concentrations in Daoy only supernatants and increased to higher levels in proportion to the degree of confluency of the Daoy cells.
(TIF)

Figure S3 **In Daoy and HBMEC cocultures, RANTES appeared anew on days 2–4 of co-culture and its levels increased rapidly 6 days after co-culture, as determined by a human RANTES-specific ELISA.**
(TIF)
