# Supplementary figures and images for: Crosstalk between Medulloblastoma Cells and Endothelium Triggers a Strong Chemotactic Signal Recruiting T Lymphocytes to the Tumor Microenvironment
Source: PLoS One. 2011 May 27;6(5):e20267. doi: 10.1371/journal.pone.0020267 (PMC3103535; doi:10.1371/journal.pone.0020267)

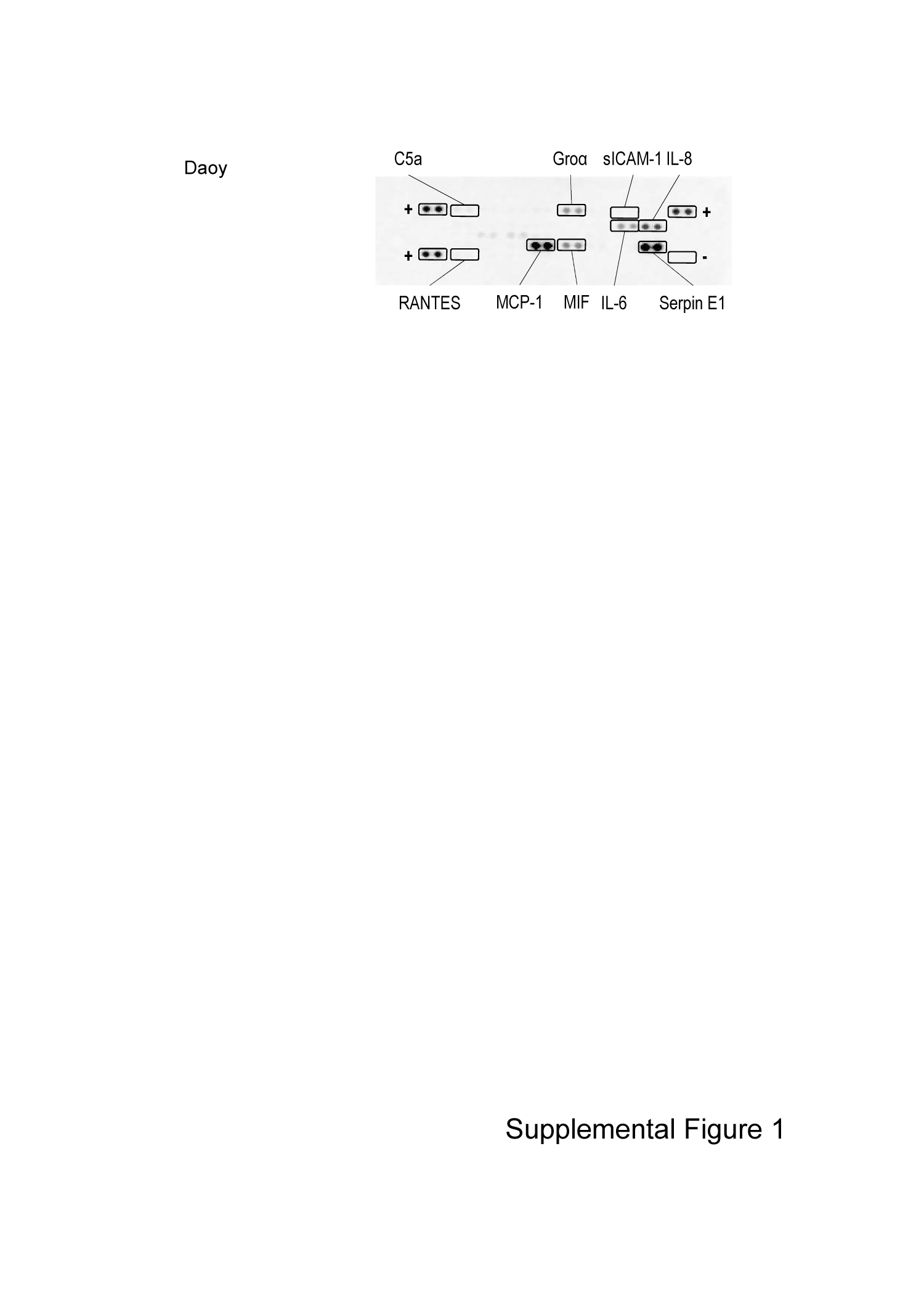

Supplement: Figure S1 — A protein profiler system was used to characterize the chemokine pattern specific to Daoy medulloblastoma cells which had a distinct chemokine signature, producing MCP-1; Gro-α; MIF; IL-8; Serpin E1; and IL-6 [quantified in Figure 3B ]. (TIF) [file pone.0020267.s001.tif]

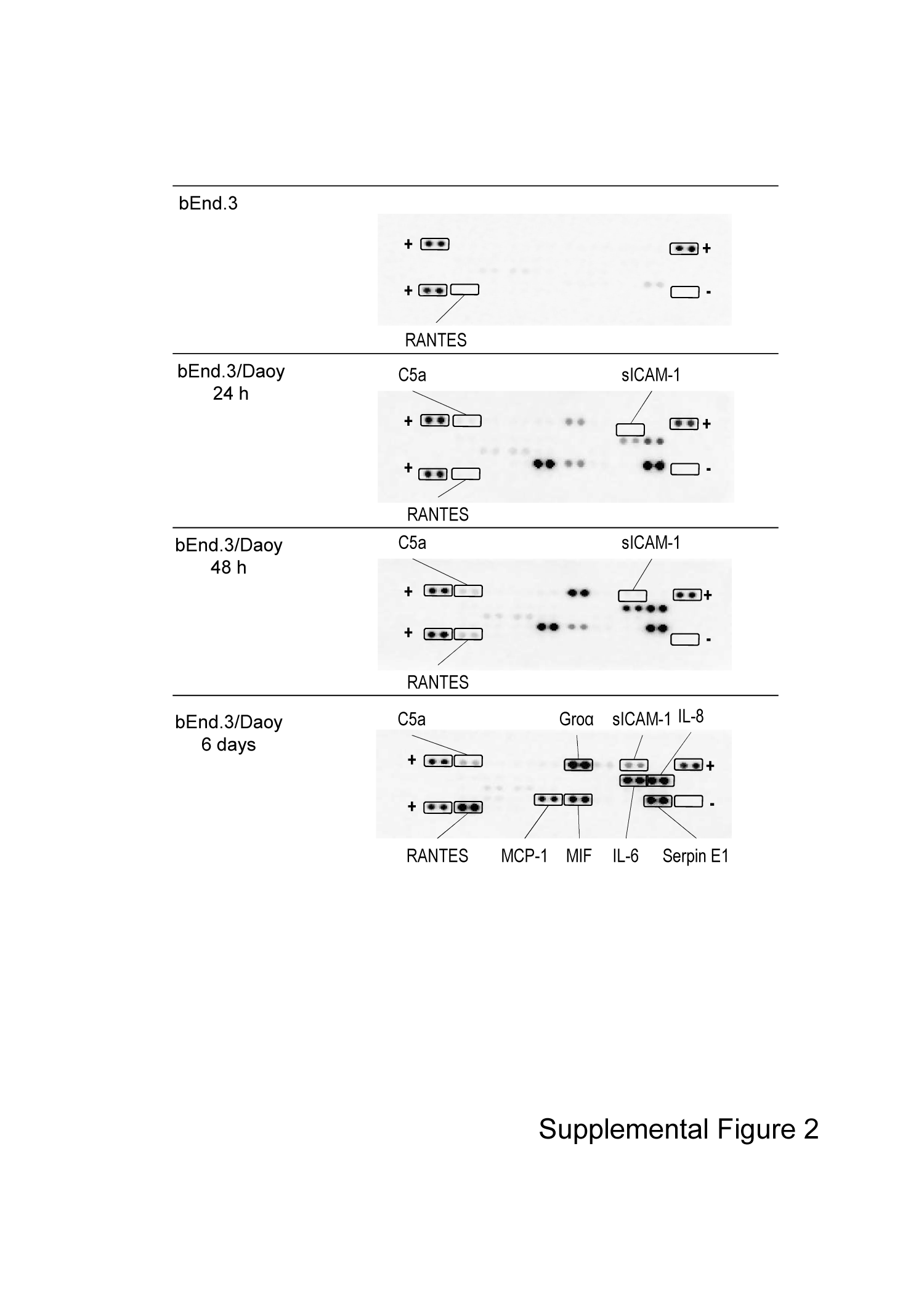

Supplement: Figure S2 — Chemokine protein profiler membranes showed that while RANTES was near undetectable in supernatant from bEND.3 or in supernatant from Daoy, its secretion substantially increased as early as 48 hours of Daoy and bEND.3 coculture and rapidly increased over time to plateau after 6 days. Other chemokines/ chemo-attractant molecules that were present in a low concentration but progressively increased in coculture were also present at low concentrations in Daoy only supernatants and increased to higher levels in proportion to the degree of confluency of the Daoy cells. (TIF) [file pone.0020267.s002.tif]

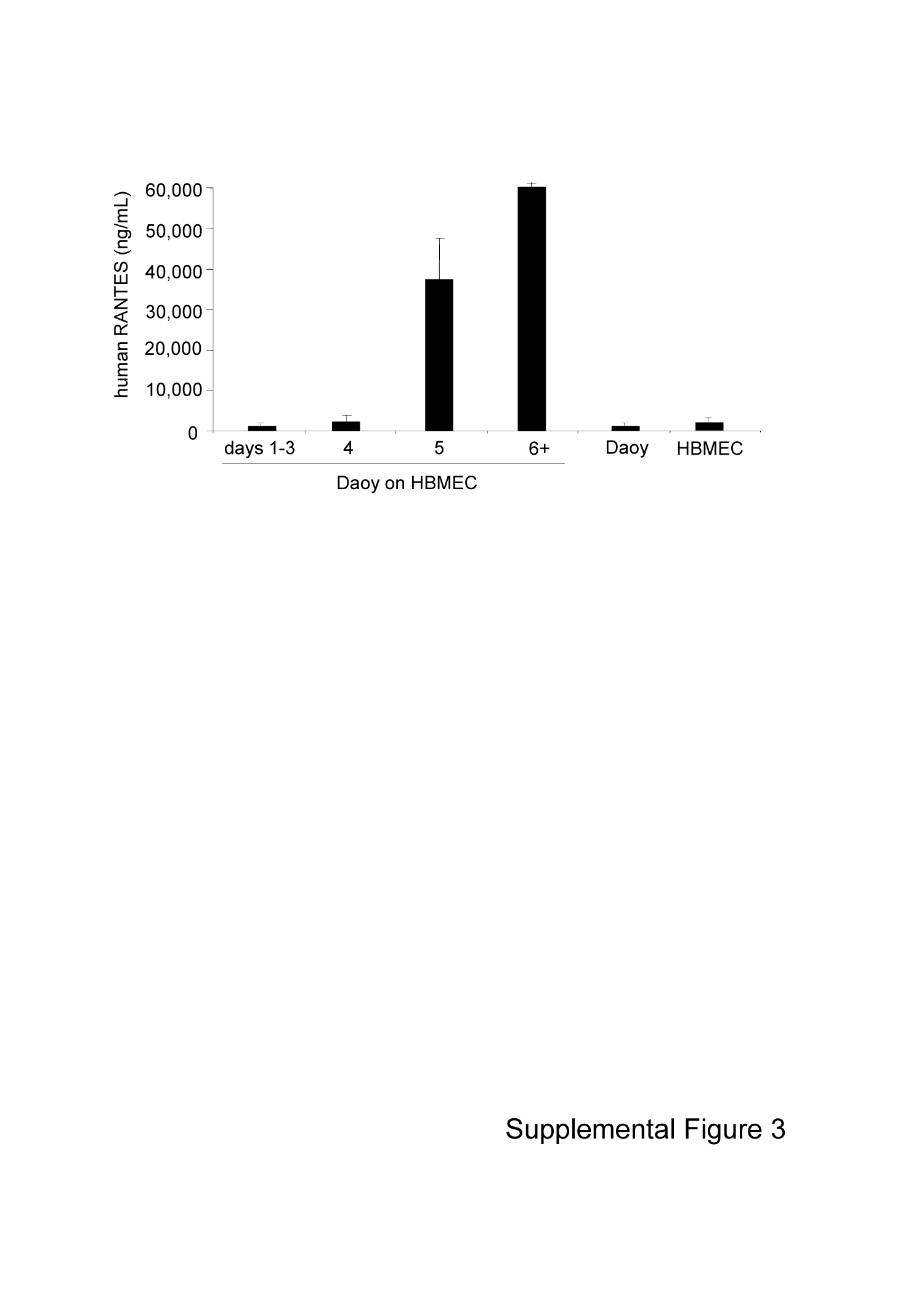

Supplement: Figure S3 — In Daoy and HBMEC cocultures, RANTES appeared anew on days 2–4 of co-culture and its levels increased rapidly 6 days after co-culture, as determined by a human RANTES-specific ELISA. (TIF) [file pone.0020267.s003.tif]
